# Vertical canopy gradients of respiration drive plant carbon budgets and leaf area index

**DOI:** 10.1111/nph.20423

**Published:** 2025-02-19

**Authors:** Jessica F. Needham, Sharmila Dey, Charles D. Koven, Rosie A. Fisher, Ryan G. Knox, Julien Lamour, Gregory Lemieux, Marcos Longo, Alistair Rogers, Jennifer Holm

**Affiliations:** ^1^ Climate & Ecosystem Sciences Division Lawrence Berkeley National Laboratory Berkeley CA 94720 USA; ^2^ Department of Earth and Planetary Sciences Harvard University Cambridge MA 02138 USA; ^3^ CICERO Center for International Climate Research 0349 Oslo Norway; ^4^ Centre de Recherche sur la Biodiversité et l'Environnement (CRBE) Université de Toulouse, CNRS, IRD, Toulouse INP, Université Toulouse 3 – Paul Sabatier (UT3) 31062 Toulouse France

**Keywords:** carbon sequestration, land surface models, leaf area index, leaf respiration, vegetation demography models

## Abstract

- Despite its importance for determining global carbon fluxes, leaf respiration remains poorly constrained in land surface models (LSMs). We tested the sensitivity of the Energy Exascale Earth System Model Land Model – Functionally Assembled Terrestrial Ecosystem Simulator (ELM‐FATES) to variation in the canopy gradients of leaf maintenance respiration (*R*_dark_).
- We ran global and point simulations varying the canopy gradient of *R*_dark_ to explore the impacts on forest structure, composition, and carbon cycling.
- In global simulations, steeper canopy gradients of *R*_dark_ lead to increased understory survival and leaf biomass. Leaf area index (LAI) increased up to 77% in tropical regions compared with the default parameterization, improving alignment with remotely sensed benchmarks. Global vegetation carbon varied from 308 Pg C to 449 Pg C across the ensemble. In tropical forest simulations, steeper gradients of *R*_dark_ had a large impact on successional dynamics.
- Results show the importance of canopy gradients in leaf traits and fluxes for determining plant carbon budgets and emergent ecosystem properties such as competitive dynamics, LAI, and vegetation carbon. The high‐model sensitivity to canopy gradients in *R*_dark_ highlights the need for more observations of how leaf traits and fluxes vary along light micro‐environments to inform critical dynamics in LSMs.

## Introduction

Autotrophic respiration is one of the three major fluxes of carbon into and out of terrestrial ecosystems. Photosynthetic assimilation of carbon dioxide (CO_2_) by plants is the largest annual global flux of CO_2_ at *c*. 123 Pg C yr^−1^, and is *c*. 20 times greater than anthropogenic CO_2_ emissions (Beer *et al*., 2010; Ciais *et al*., 2014). Together, autotrophic and heterotrophic respiration return slightly less CO_2_ back to the atmosphere. Comprehensive data on autotrophic respiration is lacking, but global fluxes are estimated at *c*. 60 Pg C yr^−1^ (Prentice *et al*., 2001). The difference between the photosynthetic and respiratory fluxes is stored by terrestrial ecosystems. In balance, terrestrial ecosystems have absorbed *c*. 20% of anthropogenic CO_2_ emissions over the last 30 yr (Le Quéré *et al*., 2018) and play a critical role in determining the rate of global change. Advancing understanding and model representation of these huge natural CO_2_ fluxes, and the balance between them, is critical for improving projections of the impacts of global change on terrestrial ecosystems, in particular for forecasting any change in the role they play in offsetting our emissions from fossil fuels (Dusenge *et al*., 2019; Friedlingstein *et al*., 2023).

About half of autotrophic respiration comes from leaves (Atkin *et al*., 2007). Yet despite its importance, model representation of leaf respiration remains a poorly constrained process in Land Surface Models (LSMs; Smith & Dukes, 2013; Lombardozzi *et al*., 2015; Huntingford *et al*., 2017; Thomas *et al*., 2019). Although recent observations offer promising leads for developing more mechanistic approaches (Bruhn *et al*., 2022; Fan *et al*., 2024), leaf maintenance respiration (*R*_dark_), CO_2_ release from the leaf associated with tissue maintenance, is typically represented in LSMs as a function of either leaf nitrogen content or of the maximum capacity for carboxylation by the enzyme Rubisco (*V*_cmax_; Thomas *et al*., 2019) and the response and acclimation of *R*_dark_ to temperature often follows that for photosynthetic parameters. Following (Farquhar *et al*., 1980) *R*_dark_ is typically assumed to be 1–2% of *V*_cmax_ across many models (Foley *et al*., 1996; Clark *et al*., 2011; Longo *et al*., 2019; Lawrence *et al*., 2020).

LSMs increasingly incorporate Vegetation Demography Models (VDMs) with multi‐layered canopies (Fisher *et al*., 2018), and the resulting vertically discretized calculation of leaf gas exchange necessitates an improved understanding of the scaling of *R*_dark_ measurements from the leaf to the canopy. Measurements of *R*_dark_, are typically taken at the top of the canopy, yet light levels vary dramatically from the top of canopy to the understory, and leaf traits and resulting fluxes change in concert (Griffin *et al*., 2001, 2002; Lloyd *et al*., 2010; Niinemets *et al*., 2015). Understanding and model representation of canopy gradients in physiological processes are both limited by datasets that describe gradients in key traits and fluxes. Typically LSMs normalize *R*_dark_ to a reference temperature (25°C, *R*_dark25_) and then scale *R*_dark25_ proportionally with maximum carboxylation capacity at the same reference temperature (*V*_cmax25_) using constant ratios (Krinner *et al*., 2005; Clark *et al*., 2011; Lawrence *et al*., 2019) implying that the vertical exponential decrease constant (*k*_*n*_; Lloyd *et al*., 2010) is the same for all vertically scaled parameters.

However, empirical work suggests that the temperature dependency and vertical canopy gradients of *R*_dark_ and *V*_cmax_ may differ. Insight into how *R*_dark_ varies across species and climate was provided by analyses of the GlobResp database, which contains measurements of *R*_dark_ from 899 species from 100 sites ranging from the Arctic to the tropics (Atkin *et al*., 2015). These analyses showed that *R*_dark_ decreases with growth temperature but that when expressed at a common reference temperature, Arctic plants have *R*_dark_ rates three times those of tropical plants (Atkin *et al*., 2015). Incorporating temperature dependencies from the GlobResp database into the LSM JULES (Joint UK Land Environment Simulator; Best *et al*., 2011; Clark *et al*., 2011) resulted in an increase in whole plant respiration by 30% globally (Huntingford *et al*., 2017). Including temperature acclimation in both photosynthesis and leaf respiration increased the terrestrial carbon pool by *c*. 20 PgC by 2100 in the Community Earth System Model, whereas photosynthetic acclimation alone resulted in an increase of *c*. 11 Pg C (Lombardozzi *et al*., 2015). This work emphasized the remaining uncertainty in model representation of *R*_dark_ and its impact on global carbon fluxes and pools.

Field observations suggest large decreases in *R*_dark25_ with canopy depth. Souza *et al*. (2021) found the mean rate of *R*_dark25_ in understory trees to be half that of canopy trees in the Amazon Forest. Critically, recent empirical work in a Panamanian tropical forest found that *R*_dark25_ decreases more rapidly than *V*_cmax25_ through the canopy, with the ratio of *R*_dark25_ to *V*_cmax25_ declining from 0.015 at the top of the canopy to 0.008 at the bottom of the canopy, an *c*. 50% decrease (Lamour *et al*., 2023a). Similar results were found in an Australian tropical forest, where *R*_dark25_ was 34% higher in the canopy than in the understory, and the ratio of *R*_dark25_ to photosynthesis was lower in understory leaves (Weerasinghe *et al*., 2014). These findings suggest a mechanism for persistence of understory trees in low‐light conditions and can inform leaf to canopy scaling in LSMs.

Optimality hypotheses assume that plants maximize the net carbon export from their canopies by adjusting physiological rates in response to environmental conditions (McMurtrie & Dewar, 2011). Optimization schemes in LSMs are sensitive to the representation of *R*_dark_, with different LAIs and leaf nitrogen values being optimal for canopy carbon export, depending on modeled *R*_dark_ (Thomas *et al*., 2019). In FATES, growth occurs daily, as plants divide up the net carbon gained over the day (photosynthesis minus growth and maintenance respiration; NPP) to various tissues as governed by allometric relationships. Allocation of carbon to leaves is dynamically adjusted based on an optimality framework in order to maximize net canopy carbon export (Fisher *et al*., 2015), by preventing allocation to leaf layers that are in annual net negative carbon balance (when the sum of leaf construction costs plus respiration exceed photosynthetic uptake), see ‘Net canopy carbon export and allocation to leaf biomass’ in the Description section. We thus expect that addition of leaf layers will be sensitive to the vertical scaling of *R*_dark_, with steeper gradients of *R*_dark_ reducing the likelihood of net negative carbon balance for leaves deeper in the canopy. We also expect understory mortality rates to decrease with steeper gradients of *R*_dark_, as understory cohorts maintain an overall more positive carbon balance. As a result of both increased survival of understory cohorts, and increased allocation of carbon to leaf biomass, the vertical scaling of *R*_dark_ could influence simulated leaf area index (LAI), with implications for transpiration, precipitation interception and latent heat fluxes (Swann *et al*., 2016).

Here, we test the effect of varying the canopy gradient of *R*_dark_ on vegetation dynamics in the ELM‐FATES (Energy Exascale Earth System Model (E3SM) Land Model – Functionally Assembled Terrestrial Ecosystem Simulator). We explore how altering the ratio between *R*_dark_ and *V*_cmax_ through the canopy impacts competitive dynamics between plant functional types (PFTs), changes understory survival and impacts global LAI and vegetation carbon. Global simulations enable us to explore the implications of changes to leaf respiration on vegetation dynamics at large scales, and the consequences for the terrestrial carbon cycle. Simulations at a single tropical forest site enable us to explore how gradients in leaf respiration might alter competitive dynamics between PFTs, as we can run large ensembles in which we vary PFT parameterizations, without high‐computational cost.

We test the following two hypotheses: (1) A steeper vertical gradient of *R*_dark_ will result in higher LAI due to an increase in the number of leaf layers in positive carbon balance, and increased understory survival. (2) In simulations with competing PFTs, a steeper vertical gradient of *R*_dark_ will favor shade‐tolerant PFTs by increasing survival in the understory.

## Description

FATES is a VDM that interfaces with a host LSM, currently ELM (Ricciuto *et al*., 2018), and CLM (the Community Land Model; Lawrence *et al*., 2019). FATES represents cohorts of individual trees that are the same size and PFT. These cohorts compete with each other on patches defined by their age since the last disturbance (Fisher *et al*., 2015; Fates Development Team, 2020; Holm *et al*., 2020; Koven *et al*., 2020). The explicit representation of canopy and understory cohorts requires scaling photosynthesis and respiration through the canopy. In FATES, leaf nitrogen decreases through the canopy following Lloyd *et al*. (2010) who used observations of 204 Amazonian trees to relate the vertical gradient of photosynthetic capacity to top of the canopy photosynthetic capacity. In the default parameterization of FATES, both *V*_cmax25_ and *R*_dark25_ are linearly related to this nitrogen scaler, resulting in a fixed ratio of *V*_cmax25_ to *R*_dark25_ with canopy depth. It is worth noting that the ratio of *V*_cmax_ to *R*_dark_ is not fixed due to their different temperature dependencies, based on Oleson *et al*. (2013) and Ryan (1991) or Atkin *et al*. (2017), respectively.

### Leaf maintenance respiration in FATES

*R*_dark_ in FATES is calculated for a PFT by leaf layer combination based on either (Atkin *et al*., 2017) or (Ryan, 1991) and is then scaled to the cohort level. In this work, we introduce in FATES the Atkin *et al*. (2017) respiration scheme, which is based on top of canopy measurements of *R*_dark_ from the GlobResp database (Atkin *et al*., 2015). These observations have been parameterized in Atkin *et al*. (2017) such that *R*_dark_ scales linearly with top of canopy leaf nitrogen content via parameter *r*_1_, with a PFT‐specific offset parameter *r*_0_, Eqn 1. Both instantaneous and acclimated temperature responses are accounted for (Heskel *et al*., 2016; Huntingford *et al*., 2017).

(Eqn 1) $$R_{\text{dark},\mathrm{top}}=r_{0}+\left(r_{1}\times {\mathrm{lnc}}_{\mathrm{top}}\right)+\left(r_{2}\times t_{10}\right)$$

*R*_dark,top_ is dark respiration at the canopy top in μmol CO_2_ m^−2^ s^−1^, *r*_0_ is a PFT‐specific base respiration rate term, lnc_top_ is leaf nitrogen content (gN m^−2^) at the top of the canopy, *r*_1_ determines respiration sensitivity to nitrogen, *t*_10_ (°C) is the running mean from a moving window of temperature over the last 10 days, and *r*_2_ determines respiration sensitivity to *t*_10_. We use *r*_1_ = 0.2061 and *r*_2_ = −0.0402 and PFT‐specific values of *r*_0_, as reported in Atkin *et al*. (2017). *R*_dark,top_ is further adjusted to account for instantaneous temperature acclimation following Heskel *et al*. (2016) using a decelerating function such that the sensitivity of respiration to temperature declines at high temperatures.

*R*_dark,top_ is scaled through the canopy using a canopy extinction coefficient from Lloyd *et al*. (2010)

(Eqn 2) $$R_{\text{dark}}=\text{nscaler}\times R_{\text{dark},\mathrm{top}}$$

where nscaler is derived from photosynthetic capacity.

(Eqn 3) $$\text{nscaler}=\exp\left(-k_{n}\times \mathrm{LAI}\right)$$

(Eqn 4) $$k_{n}=\exp\left(a\times V_{\text{cmax},25,\mathrm{top}}-b\right)$$

*k*_*n*_ is the canopy extinction coefficient, *V*_cmax,25,top_ is the maximum Rubisco activity at the canopy top at 25°C, and LAI is leaf area index. We use *a* = 0.00963 and *b* = 2.43 from Lloyd *et al*. (2010). *V*_cmax,25,top_ varies by PFT.

### Net canopy carbon export and allocation to leaf biomass

Cohorts in FATES dynamically adjust allocation of carbon to leaf biomass through a canopy‐trimming algorithm in order to maximize net canopy carbon export. Each year, the net carbon export of each leaf layer in a cohort is calculated as net annual assimilation rate minus growth and maintenance respiration and the construction cost of growing the leaves. Leaf construction costs are a function of leaf life span and specific leaf area. If net carbon export is negative, then the trimming algorithm adjusts the cohort's allometric relationship between dbh and leaf biomass. As a result, during daily allocation of NPP (GPP minus total plant respiration) the cohort will allocate less carbon to leaf biomass and instead prioritize other tissues. The reduction of leaf biomass while keeping the crown area allometry constant results in a decrease in cohort LAI. Adjusting *R*_dark_ is expected to influence the leaf layer at which net carbon export becomes negative.

### Model experiment set up

#### Global simulations

**Fig. 1:**
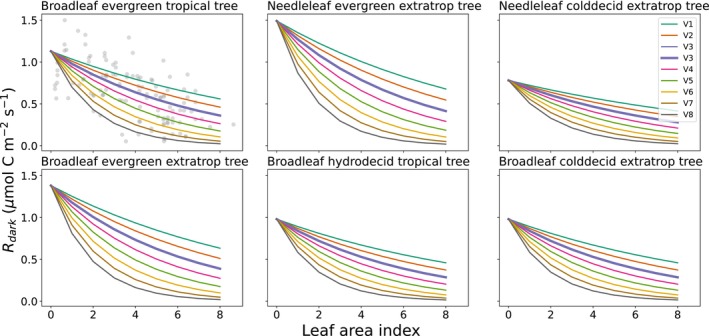
Vertical gradient of *R*_dark_ for six plant functional types (PFTs). V3 (thicker line) represents the default parameterization of the leaf maintenance respiration (*R*_dark_) canopy gradient in Functionally Assembled Terrestrial Ecosystem Simulator (FATES). Alternate vertical gradients result from changing the *b* parameter in Eqn 4; V1 = 2.916, V2 = 2.673, V3 = 2.43, V4 = 2.187, V5 = 1.944, V6 = 1.701, V7 = 1.458, V8 = 1.215. Each panel is a PFT as indicated by the panel title. Top of canopy *R*_dark_ and the gradient both depend on PFT‐specific *V*_cmax25,top_ (maximum rate of carboxylation at top of canopy at 25°C) and r_0_ parameters. Gray points in the broadleaf evergreen tropical tree panel show data from Lamour *et al.* (2023a).

We tested the sensitivity of FATES to the vertical gradient of *R*_dark_ by changing parameter *b* from Eqn 4. We varied *b* in Eqn 4 from 1.2× to 0.5× the default value in steps of 0.1 (Fig. 1). Smaller values of *b* result in steeper gradients of *R*_dark_. V1 is the simulation with the least steep gradient in *R*_dark_, while V8 has the steepest decrease in *R*_dark_ with canopy depth, with *R*_dark_ approaching 0 at an LAI of *c*. 7–8 depending on PFT. Using the linear model in Lamour *et al*. (2023a) *R*_dark25_ reaches zero at an LAI of 10; we use the exponential form here to avoid the possibility of respiration actually going to zero at that point (the Lamour *et al*. 2023a model being empirical and not intended to work outside of the LAI range observed). We kept the vertical scaling of *V*_cmax_ the same in all simulations, only varying the gradient of *R*_dark_, thus altering the ratio between them. V3 is the default gradient in which *R*_dark_ remains proportional to the carboxylation rate through the canopy. In Lamour *et al*. (2023a) the ratio between *R*_dark25_ and *V*_cmax25_ decreased by 50% from the top to the bottom of the canopy, varying from 0.015 to 0.008. Due to the parameterization of the tropical PFT in FATES, the ratio of *R*_dark_ to *V*_cmax_ was 0.023 at the top of the canopy in all simulations. In the V5 simulation, the ratio decreased by *c*. 50%, but the V6 simulation had a ratio between *R*_dark_ and *V*_cmax_ at the ground that was closest to observations (0.007). Since our hypotheses focus on how a lower *R*_dark_ to *V*_cmax_ ratio at the bottom of the canopy impacts understory leaf carbon allocation and understory survival, we refer to the V6 simulation as the most observationally constrained.

For this experiment we ran FATES in the reduced complexity configuration ‘fixed biogeography, no competition mode’ (hereafter ‘nocomp mode’), in which PFT distributions are prescribed based on satellite observations, and individual PFTs occupy distinct patches. Within a patch, cohorts of different sizes compete with one another for light and water, resulting in size‐structured competition within a given PFT, but no competition among PFTs. Nocomp mode includes all the physiological mechanisms of full complexity FATES, allowing us to test the effects of altered respiration on leaf allocation and stand structure, while controlling for competitive dynamics between PFTs. A full description of nocomp mode and other reduced complexity configurations is in the FATES technical note (https://fates‐users‐guide.readthedocs.io/projects/tech‐doc/en/stable/). For each simulation with a different value of the *b* parameter, we spun up the simulation from bare ground for 290 yr and then used a 10 yr simulation to assess the impacts of vertical gradients of *R*_dark_.

In global simulations we used a parameter file with the default 12 global PFTs, with some initial calibration of parameters related to GPP, albedo, ET, and allometry, see Needham (2025). We increased the maximum number of canopy layers to three, to prevent any restriction on the number of understory cohorts. We only varied *b* for the six tree PFTs, Fig. 1. We ran at a 4 × 5 degree resolution with no harvest or transient land use change, and treated croplands as areas occupied by grass PFTs.

#### Single site simulations

To test the effects of different vertical gradients of *R*_dark_ on PFT coexistence we ran simulations at a single site with a light demanding and shade‐tolerant PFT in full complexity FATES in which PFTs can compete against each other on any given patch. We used climate‐forcing data from drivers measured at the Barro Colorado Island meteorological station between 1986–2017, with data processing described in Faybishenko *et al*. (2018). We first ran an ensemble of simulations using the default gradient of *R*_dark_ to identify parameter sets with reasonable aboveground biomass (AGB), and that represented the three cases of either the shade‐tolerant PFT dominating in terms of AGB, the light demanding PFT dominating, or some degree of coexistence between PFTs after 100 yr. Parameters were sampled using latin hypercube sampling (LHS) with some fixed relationships between PFT values (see Supporting Information Table S1). We used top of canopy *V*_cmax25_ from the Lamour *et al*. (2023a) dataset as the value for the light demanding PFT and allowed the top of canopy *V*_cmax25_ for the shade‐tolerant PFT to be sampled as part of the LHS. Parameter sampling was also constrained so that the light demanding PFT had a higher base rate of *R*_dark_. We tested the sensitivity of PFT coexistence to the vertical gradient of *R*_dark_ by again changing parameter *b* from Eqn 4 for both PFTs. In single site simulations we ran four alternate scalings, corresponding to V1 (highest understory respiration), V3 (default canopy gradient in *R*_dark_), V5 and V7 (steeper canopy gradients in *R*_dark_) from the global simulations. We ran simulations from bare ground for 300 yr.

## Results

In global simulations, we find that steeper vertical gradients of *R*_dark_ result in greater carbon allocation to leaf biomass, understory canopy cover, and LAI. In the simulation with the steepest gradient, LAI is 41% higher globally compared to the default parameterization. In single site simulations, steeper vertical gradients of *R*_dark_ favored the light demanding PFT and in some cases reversed the dominance of PFTs.

### Global simulations

#### Effects on leaf allocation

As expected, steeper canopy gradients of *R*_dark_ allow leaf layers lower in the canopy to remain in positive carbon balance, leading to greater allocation of NPP to leaf biomass, see ‘Net canopy carbon export and allocation to leaf biomass’ in the Description section. In the V8 simulation with the steepest gradient in *R*_dark_, allocation of carbon to leaf biomass in the Amazon ecoregion (based on Olson *et al*., 2001) increased by 32% relative to the default V3 simulation, from 0.056 kg C m^−2^ yr^−1^ to 0.074 kg C m^−2^ yr^−1^. The additional leaves were primarily in the understory; allocation of NPP to leaf biomass increased by 105% in the understory and 16% in the canopy, in the V8 simulation relative to the V3 simulation across the Amazon ecoregion (Figs S1, S2).

#### Effects on understory dynamics

**Fig. 2:**
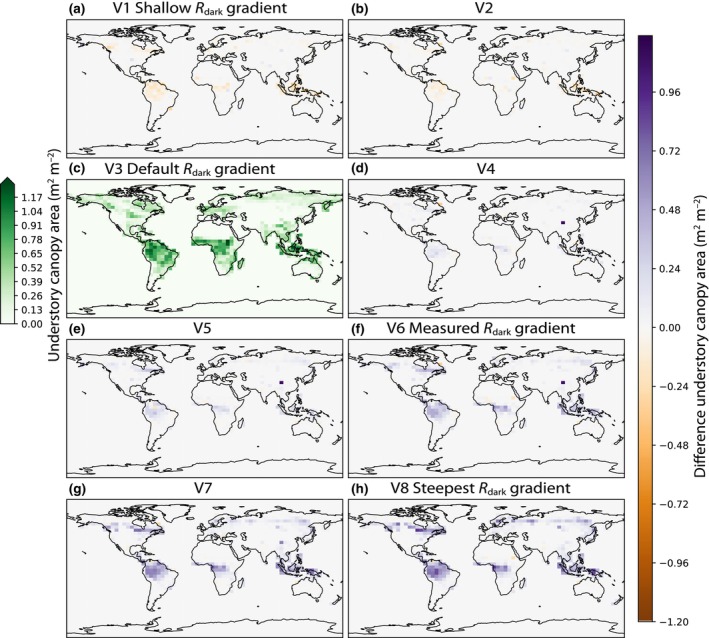
Effects of *R*_dark_ vertical gradients on understory canopy area in Functionally Assembled Terrestrial Ecosystem Simulator (FATES). V3 is the default simulation in FATES in which leaf maintenance respiration (*R*_dark_) and maximum rate of carboxylation (*V*_cmax_) are proportional through the canopy. The V3 panel shows the absolute values of the understory canopy area. Remaining panels show the difference between a given simulation and the default (corresponding to colored lines in Fig. 1). (a) V1 and (b) V2 have less steep gradients of *R*_dark_ compared with the default (c). The canopy gradients of *R*_dark_ in (d) V4, (e) V5, (f) V6, (g) V7, and (h) V8 get progressively steeper. V6 is the simulation in which the ratio of *R*_dark_ to *V*_cmax_ at the ground is closest to observations from Lamour *et al*. (2023a). Purple indicates that a given simulation had a higher understory canopy area than the default (V3), and orange indicates that a given simulation had a lower understory canopy area than the default (V3).

Steeper gradients of *R*_dark_ resulted in more numerous understory plants and a more complete understory canopy layer (Fig. 2). In the V3 simulation, with the default canopy gradient, the understory canopy layer is not fully closed (defined as 1 m^2^ canopy area per m^−2^ ground area) even in tropical forests. Across the Amazon ecoregion, mean understory canopy area increased from 0.74 m^2^ m^−2^ in the V3 simulation with the default parameterization to 1.23 m^2^ m^−2^ in the V8 simulation with the steepest canopy gradient of *R*_dark_, indicating that, on average, more than one understory layer is present. In the Congo Rainforest (defined here as latitude −8.0 to 7.0 and longitude 8.0 to 32.0), understory canopy area increased from 0.70 m^2^ m^−2^ in the default V3 simulation to 1.02 m^2^ m^−2^ in the V8 simulation.

Across the Amazon ecoregion, carbon starvation mortality rates in understory trees were highest in the V1 simulation, with the highest understory respiration, at 13% yr^−1^ and decreased with steeper vertical gradients of *R*_dark_ until the V6 simulation at 9% yr^−1^. In the V7 and V8 simulation carbon starvation mortality rates increased again to 12% yr^−1^, due to limited light in the understory. Across the Congo rainforest, understory carbon starvation mortality rates were highest at 12% yr^−1^ in the V1 simulation with the least steep gradient of *R*_dark_, lowest at 8% yr^−1^ in the V6 simulation and again increased in the V8 simulation with the steepest gradient of *R*_dark_, to 10% yr^−1^.

#### Effects on LAI

**Fig. 3:**
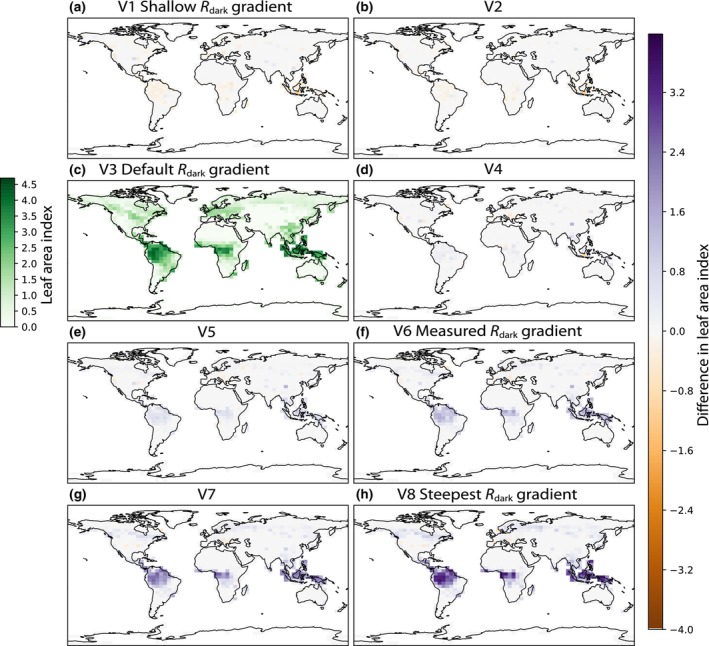
Effects of *R*_dark_ vertical gradients on Leaf Area Index in Functionally Assembled Terrestrial Ecosystem Simulator (FATES). V3 is the default simulation in FATES in which leaf maintenance respiration (*R*_dark_) and maximum rate of carboxylation (*V*_cmax_) are proportional through the canopy. The V3 panel shows the absolute values of leaf area index (LAI). Remaining panels show the difference between a given simulation and the default (corresponding to colored lines in Fig. 1). (a) V1 and (b) V2 have less steep gradients of *R*_dark_ compared with the default (c). The canopy gradients of *R*_dark_ in (d) V4, (e) V5, (f) V6, (g) V7, and (h) V8 get progressively steeper. Purple indicates that a given simulation had higher LAI than the default simulation (V3), whereas orange indicates lower LAI than the default simulation (V3).

In simulations with steeper vertical gradients of *R*_dark_, the increased allocation to leaf biomass, combined with an increase in understory canopy area resulted in large increases in LAI (Fig. 3). Across the ensemble (V1–V8), LAI increased by 90%, from 2.9 to 5.5 in the Amazon ecoregion, and by 71% from 2.4 to 4.1 in the Congo rainforest. In South East Asia (defined here as latitude −11.0 to 20.0 and longitude 90.0 to 152.0), LAI is lower due to the presence of crops, but increased by 78% across the ensemble, from 0.9 to 1.6. Again, increases in LAI were driven by understory leaf layers, with understory LAI in the Amazon ecoregion increasing from 0.9 to 3.2 across the ensemble and canopy LAI increasing from 2.0 to 2.3 (Figs S3, S4).

#### Effects on vegetation carbon

**Fig. 4:**
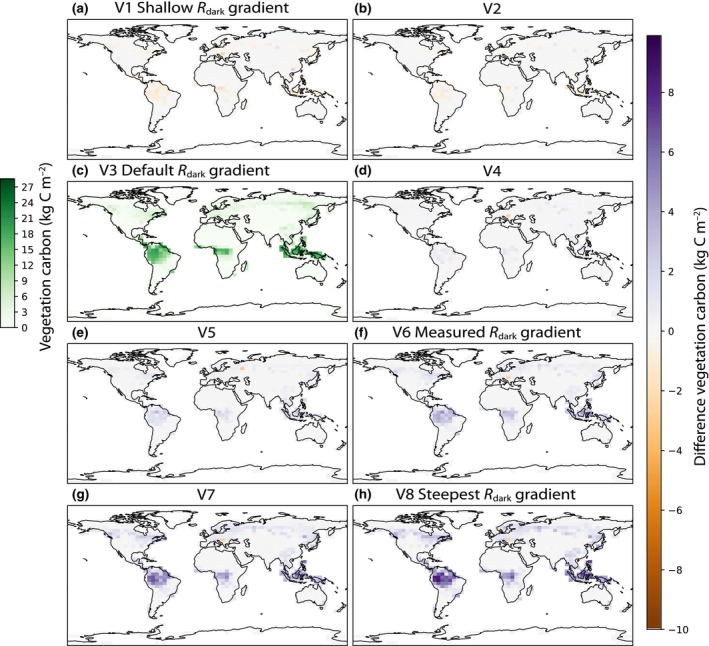
Effects of *R*_dark_ vertical gradients on vegetation carbon in Functionally Assembled Terrestrial Ecosystem Simulator (FATES). V3 is the default simulation in FATES in which leaf maintenance respiration (*R*_dark_) and maximum rate of carboxylation (*V*_cmax_) are proportional through the canopy. The V3 panel shows the absolute values of vegetation carbon, the total carbon in both above‐ and belowground plant tissues. Remaining panels show the difference between a given simulation and the default (V3). (a) V1 and (b) V2 have less steep gradients of *R*_dark_ compared with the default (c). The canopy gradients of *R*_dark_ in (d) V4, (e) V5, (f) V6, (g) V7, and (h) V8 get progressively steeper. Purple indicates that a given simulation had higher vegetation carbon than the default simulation (V3), whereas orange indicates lower vegetation carbon than the default simulation (V3).

The amount of carbon stored in vegetation biomass increased by 39% across the Amazon ecoregion in the V8 simulation compared with the default V3 simulation (Fig. 4), from 13.3 to 18.6 kg C m^−2^. In the Congo rainforest and across South East Asia vegetation carbon increased by 31% and 29% respectively, from the default V3 to the V8 simulation. In the V1 simulation, with a shallow vertical gradient of *R*_dark_, vegetation carbon decreased by 4% across the Amazon ecoregion compared to the default, down to 12.8 kg C m^−2^. Globally, vegetation carbon increased from 308 Pg C to 449 Pg C from the V1 to V8 simulations, with the V8 simulation representing a 38% increase compared to the default simulation.

#### Global benchmarking

**Fig. 5:**
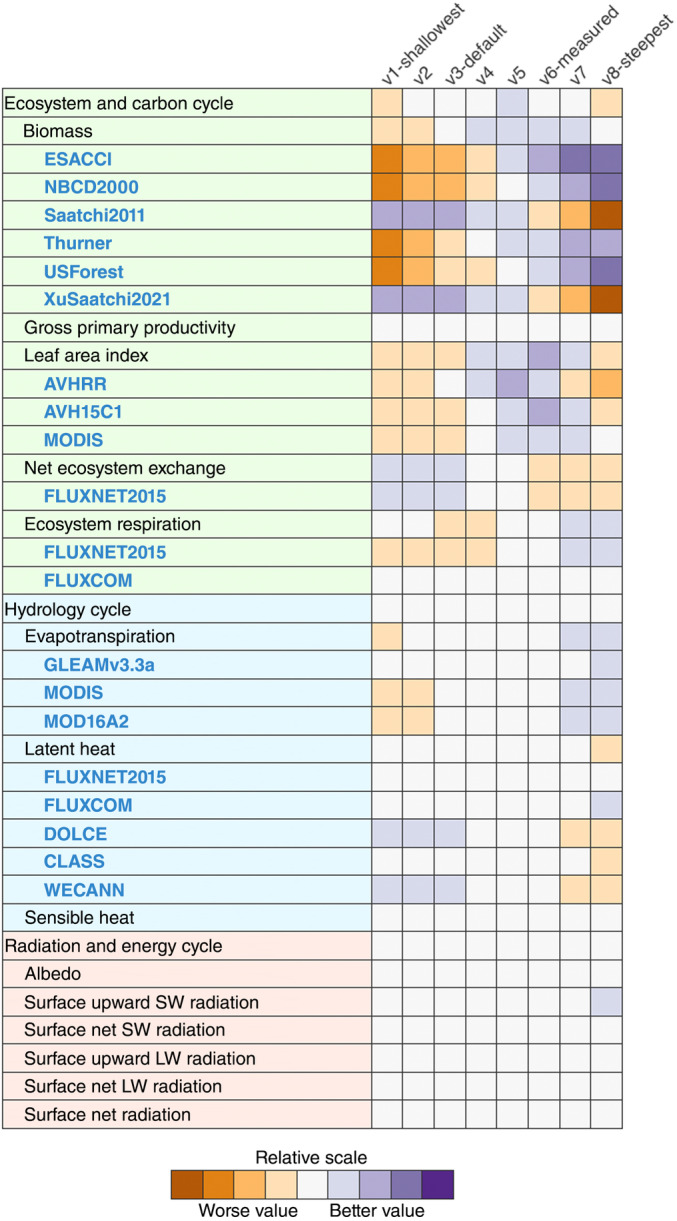
International Land Model Benchmarking project (ILAMB) comparison of vertical gradients of leaf maintenance respiration (*R*_dark_) against observational data. ILAMB columns show model simulations, with V3 being the default Functionally Assembled Terrestrial Ecosystem Simulator (FATES) parameterization. Rows show observational data sets. Colors correspond to how well the simulation matches observations, with purple indicating a better fit. The V6 simulation, with a steeper vertical gradient of *R*_dark_ and maximum rate of carboxylation (*V*_cmax_), led to the best leaf area index score. Diverging biomass scores for different datasets reflect the uncertainty in the observational benchmarks.

The International Land Model Benchmarking project (ILAMB) is a model‐data intercomparison project that performs comprehensive assessments of model simulations across observations of land variables from *in situ*, remote sensing and reanalysis datasets (Collier *et al*., 2018). For a detailed description of the ILAMB scoring methodology, see (Collier *et al*., 2018). Steeper *R*_dark_ improved comparisons of FATES LAI with data from MODIS (Moderate Resolution Imaging Spectroradiometer) and AVHRR (Advanced Very High Resolution Radiometer) both remote sensing products, up until the V6 simulation, and then comparison scores became worse (Fig. 5). Evapotranspiration, estimated from MODIS, and ecosystem respiration, from FLUXNET (Lasslop *et al*., 2010), a product based on data from networks of flux towers, also improved with steeper *R*_dark_, with the best simulations being V7 and V8 for both metrics. The biomass datasets in ILAMB diverge widely and therefore scores for FATES improved with steeper *R*_dark_ compared to ESACCI (Santoro & Cartus, 2021) and northern latitude datasets (NBCD2000, Thurner (Thurner *et al*., 2014) and USForest) but decreased for the Saatchi2011 (Saatchi *et al*., 2011) and XuSaatchi2021 (Xu *et al*., 2021) datasets. Full ILAMB results can be viewed at https://portal.nersc.gov/cfs/e3sm/jneedham/rdark_300724_paperv/.

### Single site simulations

**Fig. 6:**
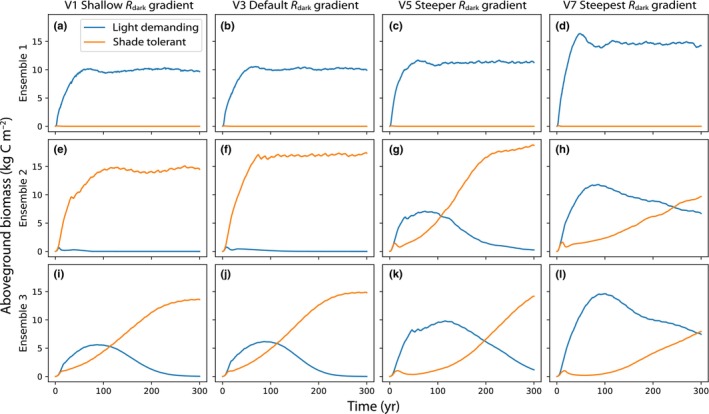
Coexistence between plant functional types (PFTs) in single site simulations using alternate vertical gradients of leaf maintenance respiration (*R*_dark_). Rows are different parameterizations of the two PFTs, in which parameters related to life history strategy were sampled using latin hypercube sampling (see Supporting Information Table S1). Columns are alternate parameterization of the *R*_dark_ vertical gradient, with a shallow gradient on the left, and the steeper gradients on the right. The default gradient, in which *R*_dark_ is proportional to carboxylation rate through the canopy, is the second column, V3. (a–d) Represents a parameterization of PFTs in which the light demanding PFT dominates with the default parameterization of the *R*_dark_ vertical gradient. (e–h) Is a PFT parameterization where the shade tolerant PFT dominates with the default parameterization of *R*_dark_. (i–l) Is a PFT parameterization where there is coexistence after 100 years with the default parameterization of *R*_dark_.

We find that the scaling of respiration through the canopy has a large impact on coexistence of PFTs (Fig. 6). When the light demanding PFT dominated with the default canopy gradient of *R*_dark_, simulations with steeper gradients had higher biomass of the light demanding PFT, while the shade‐tolerant PFT was uncompetitive regardless of *R*_dark_ scaling. When the shade‐tolerant PFT dominated with the default canopy gradient of *R*_dark_, simulations with steeper canopy gradients had higher competitiveness of the light demanding PFT, and coexistence after 300 yr. In the final parameterization, the default scaling of *R*_dark_ resulted in the light demanding PFT initially dominating in terms of biomass, but being slowly outcompeted by the shade‐tolerant PFT over the 300 yr simulation. The simulation with steeper canopy gradients of *R*_dark_ had increased competitiveness of the light demanding PFT, delaying the point at which the shade‐tolerant PFT became more abundant by nearly 200 yr. The dynamics of these simulations are dependent on the parameterization of the two PFTs, but suggest an overall trend of steeper canopy gradients favoring light demanding PFTs.

## Discussion

Typically, LSMs assume a fixed ratio between *V*_cmax25_ and *R*_dark25_, but recent observations showed that *R*_dark25_ decreases to a greater extent than *V*_cmax25_ from the top of a forest canopy to the understory (Weerasinghe *et al*., 2014; Lamour *et al*., 2023a). We investigated how different canopy gradients of *R*_dark_ impact vegetation dynamics in the vegetation demography model ELM‐FATES. We varied the scaling of *R*_dark_ from the top to the bottom of the canopy, while holding the vertical canopy gradient of *V*_cmax_ constant, therefore changing the ratio between them. We find that steeper canopy gradients of *R*_dark_ can increase forest LAI through increased allocation to leaf biomass and increased understory survival. Our results suggest that plant metabolism has an important role in determining LAI in forests, which has cascading effects on GPP, vegetation carbon and forest structure. This analysis highlights the importance of improving model representation of physiological processes, their scaling with canopy depth, and their impact on global carbon fluxes and storage.

**Fig. 7:**
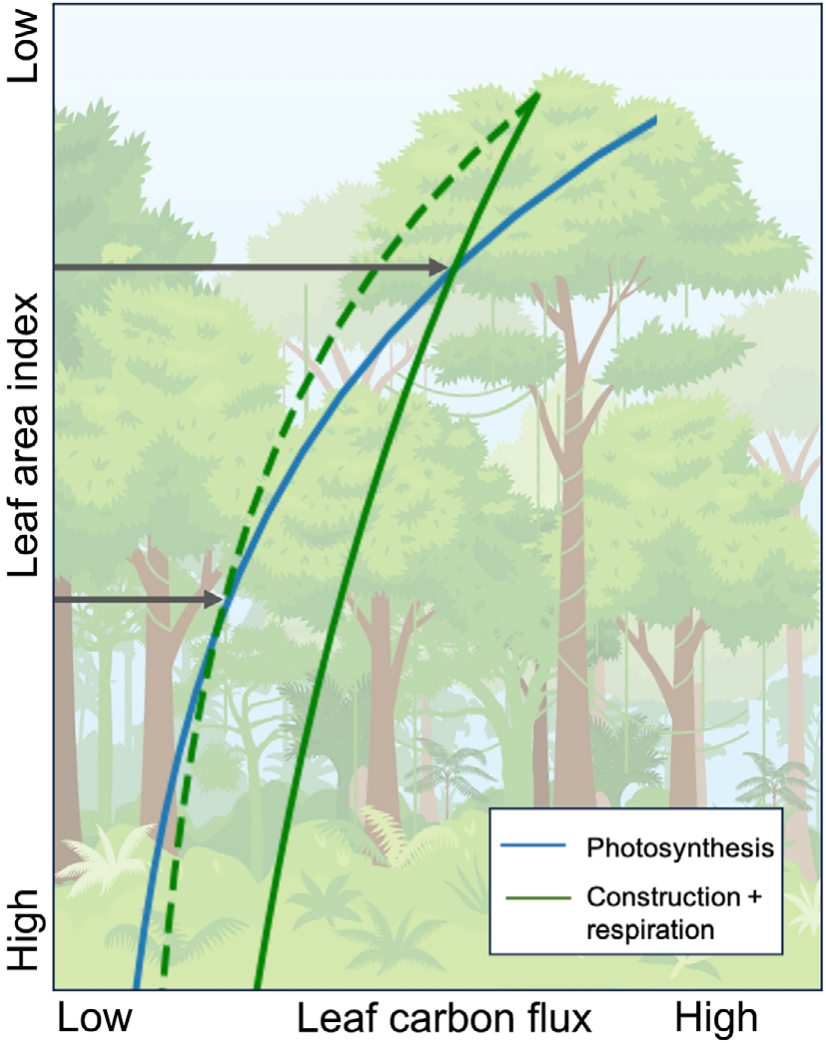
Schematic of leaf layer carbon balance. Allocation to leaf biomass is dynamic so that leaf layers are only grown and maintained when photosynthesis is greater than the combined cost of leaf construction and respiration. As the canopy gradient of leaf maintenance respiration (*R*_dark_) becomes steeper (dashed line), the point at which photosynthesis is less than leaf construction and respiration, that is the leaf area index of maximum net canopy carbon export, moves lower in the canopy.

The LAI is partly determined by the balance between photosynthesis and respiration through the canopy. Photosynthesis declines through the canopy because there is less light available for CO_2_ assimilation and a decreasing investment in photosynthetic machinery (Niinemets *et al*., 2015). One hypothesis for the concurrent decline in *R*_dark_ is that there is less respiratory cost associated with the photosynthetic machinery to maintain, and also the requirement for energy intensive repair of photodamaged proteins decreases with the lower light and photosynthetic activity in the understory (Murata & Nishiyama, 2018; Vinod *et al*., 2023). In FATES, plants dynamically adjust allocation to leaf biomass to optimize net canopy carbon export, in other words, they do not grow leaf layers where the annual respiratory costs of leaf construction and maintenance would outweigh the carbon fixed (see ‘Net canopy carbon export and allocation to leaf biomass’ in the Description section). In line with our first hypothesis, we found that as the canopy gradient of *R*_dark_ becomes steeper, the point at which photosynthesis is less than construction costs plus respiration, that is the LAI of maximum net canopy carbon export (Thomas *et al*., 2019), moves lower in the canopy (Fig. 7).

Additional leaf layers are associated with greater growth respiration in simulations with steeper canopy gradients of *R*_dark_. Similar to other LSMS, growth respiration in ELM‐FATES is a constant across PFTs and tissue types (Krinner *et al*., 2005; Clark *et al*., 2011; Ziehn *et al*., 2011) calculated as 0.11 multiplied by GPP minus total plant maintenance respiration. Overall, growth respiration is relatively small compared to *R*_dark_ (Fig. S5), and the greater growth respiration in simulations with steeper gradients of *R*_dark_ (Fig. S6) is outweighed by lower maintenance respiration (Fig. S7) and greater GPP. In reality, growth and maintenance respiration are not distinct processes, and the respiratory costs associated with growth are likely to vary across species and tissue types, and in response to environmental conditions. A more complete and mechanistic implementation of growth respiration in LSMs would require data on how energy requirements for biosynthesis vary across species and environmental gradients. This should be a high priority given the large uncertainty currently associated with autotrophic growth respiration (Dietze *et al*., 2014).

We found that LAI in ELM‐FATES is highly sensitive to the scaling of *R*_dark_ through the canopy and its relationship to *V*_cmax_. With the default canopy gradient of *R*_dark_, LAI was biased low in global simulations of ELM‐FATES, especially in tropical regions. LAI over the Amazon ecoregion increased from 3.1 with the default canopy gradient to 5.5 with the steepest gradient of *R*_dark_, which is in line with ground observations across the region (Negrón Juárez *et al*., 2009). Steeper canopy gradients of *R*_dark_ also improved the agreement between simulations and observations of LAI from remote sensing. From ILAMB, the V6 simulation, which has an *R*_dark_ to *V*_cmax_ ratio at the ground closest to the ratio measured in Lamour *et al*. (2023a), is best aligned overall with MODIS, AVH15C1 and AVHRR. However, it is worth noting that remote sensing observations saturate at high‐LAI values (Hilker *et al*., 2015) and ground measurements of LAI can exceed 7 or more in tropical forests (Lamour *et al*., 2023a). Understory LAI increased significantly with steeper gradients of *R*_dark_ in our simulations, leading to better agreement with LiDAR estimates of leaf area density through the vertical canopy profile that show significant leaf biomass in the lower 50% of the canopy (Stark *et al*., 2015).

Leaf area has an important impact on the exchange of water, energy and carbon between the land and atmosphere. LAI determines transpiration and evapotranspiration rates. Transpiration fluxes are a significant portion of latent heat flux over land, particularly in the tropics (Wei *et al*., 2017), while in coupled simulations, changes in evapotranspiration can feedback and alter global precipitation (Kooperman *et al*., 2018). Further, high LAI due to growth under favorable climate conditions increases potential evapotranspiration and may make forests more vulnerable to drought in years with hot and dry conditions, a process known as structural overshoot. Zhang *et al*. (2021) found structural overshoot contributed to 11% of global droughts between 1981 and 2015. It is therefore critical that LSMs represent the processes that determine LAI and understand how these may change under future climatic conditions.

We find that higher LAI increases total GPP, and combined with lower *R*_dark_ leads to an increase in carbon use efficiency, net primary productivity and vegetation carbon. Globally, GPP increased from 107 to 117 Pg C yr^−1^ from the least to most steep canopy gradient in *R*_dark_. Direct observations of GPP do not exist globally, and there is large uncertainty in modeled GPP. A light use efficiency model driven by remote sensing observations estimates global GPP at 130 ± 1.6 Pg C yr^−1^ (Madani *et al*., 2020), while across the TRENDY consortium of global dynamic vegetation models GPP is estimated between 130–169 Pg C yr^−1^ (Anav *et al*., 2015). Even the highest GPP value, resulting from the steepest gradients in *R*_dark_, is low compared to these estimates. However, we note here that we have not made an attempt to formally calibrate model parameters against global GPP products, thus the absolute value of GPP is less relevant than its high sensitivity to the vertical profile in respiration, in which about a quarter of the TRENDY ensemble spread in GPP could be generated with this one poorly constrained process.

Increases in GPP across our ensemble were smaller than increases in net primary productivity (NPP) leading to higher carbon use efficiency (NPP/GPP) with steeper canopy gradients of *R*_dark_. Over the Amazon ecoregion, carbon use efficiency was 0.48 in the simulation with the steepest decrease in *R*_dark_. This is high compared with other estimates of carbon use efficiency (Zhang *et al*., 2014), but the bias might be due to very low‐stem respiration in our simulations. Stem respiration is poorly constrained by observations, and has been set to near zero in the default parameterization of FATES to prevent very high‐carbon starvation mortality of larger trees. Future work will test whether the combination of stem respiration and steeper canopy gradients of *R*_dark_ allow for reasonable carbon use efficiency without causing vegetation dieback. Changes to GPP and carbon use efficiency led to total global vegetation carbon varying from 308 to 449 Pg C across the ensemble of *R*_dark_ scaling. Xu *et al*. (2021) estimated global vegetation carbon to be *c*. 381 Pg C as of 2019, closest to our V6 simulation, which had total global vegetation carbon of 384 Pg C.

As expected, understory carbon starvation mortality mostly decreased with steeper canopy gradients of *R*_dark_ as plants remained in more positive carbon balance. However, with the steepest decreases in *R*_dark_ in the V8 simulation, understory carbon starvation mortality increased and was higher than in the default simulation in the Amazon ecoregion, the Congo rainforest and in Southeast Asian forests. This suggests a negative feedback mechanism through which reduced *R*_dark_ in the understory leads to increases in LAI, which reduces light in the understory. At some point, light becomes so limiting that even with reduced *R*_dark_, understory plants are unable to photosynthesize enough to remain in positive carbon balance, they eventually die of carbon starvation, and the ecosystem reaches the LAI upper limit. Our results suggest that *R*_dark_ can decrease significantly more than *V*_cmax_ and thus increase the upper limit of LAI. Further, even with increased carbon starvation mortality, the steepest canopy gradients in *R*_dark_ still led to the highest LAI and understory canopy cover.

Observations have shown that understory trees can survive for many years in shaded environments. Radiocarbon dating of trees across three sites in the Amazon found numerous understory trees (< 30 cm DBH) that were several hundred years old (Vieira *et al*., 2005). In African forests, understory trees contain relatively little biomass, yet, because of their long persistence in the understory, account for 20% of the forest carbon sink (Hubau *et al*., 2019). FATES and other VDMs have typically struggled to recreate the dynamics of understory trees, with high mortality and compensating recruitment in the understory (Hanbury‐Brown *et al*., 2022). Using more observationally constrained variation in *R*_dark_ through the canopy, simulated understory dynamics are more ecologically realistic, due to reductions in high rates of carbon starvation mortality. Understory canopy area was 0.74 m^2^ m^−2^ across the Amazon ecoregion in the default simulation indicating an incomplete understory layer. With the steepest gradient in *R*_dark_ understory canopy area increased to 1.23 m^2^ m^−2^ (and total canopy area to 2.23 m^2^ m^−2^). While this is still low compared to the estimate of 3.1 canopy layers in a Panamanian forest (Bohlman & Pacala, 2012), it represents an improvement on the default parameterization. Mortality and disturbance parameters are at present largely unconstrained in global simulations of FATES. Future work will test the impact of canopy gradients in respiration on understory and total vegetation carbon residence time in a more fully calibrated version of the model.

A key advantage of VDMs over traditional LSMs is their ability to capture competitive dynamics between PFTs and therefore simulate successional dynamics (Fisher *et al*., 2018). This is critical given the predicted increase in forest disturbance, and the growing importance of secondary forests globally (Pugh *et al*., 2019a,b). Light demanding species have high‐photosynthetic capacity in the light, but this comes at the cost of low survival in low‐light conditions, when photosynthesis cannot support the high‐respiratory costs of maintaining a large investment in photosynthetic enzymes. We therefore expected greater dominance of the shade‐tolerant PFT when canopy gradients of *R*_dark_ were steeper due to the expected increase in LAI. Contrary to expectations, we found that steeper gradients of *R*_dark_ tended to increase the dominance of light demanding PFTs. This may point to problems with the parameterization of our PFTs – noting that calibration of competition in VDMs is challenging (Koven *et al*., 2020). We set the base rate of *R*_dark_ to be higher in the light demanding PFT, but kept scaling parameters constant between PFTs. As a result, simulations with steeper gradients resulted in a relatively greater decrease in *R*_dark_ for the light demanding PFT than the shade‐tolerant PFT (Fig. S8). Canopy gradients of *R*_dark_ may need to be less steep in light demanding PFTs to cause the understory mortality observed in the field. In line with this, observational studies point to varying *R*_dark25_ to *V*_cmax25_ ratios across life history strategies (Ziegler *et al*., 2020; Lamour *et al*., 2023b).

While these results represent a step towards a more mechanistic and empirically constrained representation of autotrophic respiration in LSMs, several areas of improvement remain. Light inhibition of leaf maintenance respiration also impacts vertical gradients in leaf carbon fluxes by limiting respiration at the top of the canopy, especially in high‐light environments (Tcherkez *et al*., 2017; Heskel & Tang, 2018; Souza *et al*., 2021). *R*_light_ has been estimated to be 80% lower than *R*_dark_ in a herbaceous and woody species (Atkin *et al*., 2006; Zaragoza‐Castells *et al*., 2008). Lloyd *et al*. (2010) provide a model for light inhibition of respiration using data from *Eucalyptus pauciflora* (Atkin *et al*., 2000) which results in a 82% reduction in leaf respiration at 200 μmol photons m^−2^ s^−1^. The LSM JULES has implemented a constant 30% inhibition of leaf respiration when irradiance exceeds 10 μmol photos m^−2^ s^−1^ (Clark *et al*., 2011; Harper *et al*., 2016). In our simulations, the lack of light inhibition likely led to an overestimate of total *R*_dark_, which may also have contributed to the low LAI in the default parameterization.

Many leaf‐level traits are known to change from the top to the bottom of the canopy in response to varying light availability (Niinemets *et al*., 2015; Lamour *et al*., 2023a), yet there are little data on canopy gradients of *R*_dark_, despite the central importance of leaf respiration for determining the whole plant carbon budget (Weerasinghe *et al*., 2014; Lamour *et al*., 2023a). We have shown for the first time that global simulations of a VDM are highly sensitive to the canopy gradient of *R*_dark_ and its relationship with carboxylation. By altering the carbon budget of understory leaf layers, the canopy gradient of *R*_dark_ impacts understory survival and allocation to leaf biomass with consequences for LAI and total vegetation biomass. More data on how respiration varies with LAI and carboxylation across a range of biomes would help constrain parameterization in VDMs running regionally or globally.

## Competing interests

None declared.

## Author contributions

JFN, JH and CDK came up with the experimental design. JFN implemented alternative vertical scaling of dark respiration in FATES and ran all simulations. SD designed and analyzed BCI simulations. JFN analyzed all global simulations. RGK, GL, RAF, JH, CDK, ML and JFN are members of the core FATES development team and advised on new feature implementation in FATES and interpretation of results. JL and AR contributed data from Panama on vertical profiles of dark respiration and *V*_cmax_. JFN wrote the manuscript with contributions and feedback from all authors.

## Disclaimer

The New Phytologist Foundation remains neutral with regard to jurisdictional claims in maps and in any institutional affiliations.

## Supporting information


**Fig. S1** Effects of *R*_dark_ vertical gradients on understory leaf allocation in FATES.
**Fig. S2** Effects of *R*_dark_ vertical gradients on canopy leaf allocation in FATES.
**Fig. S3** Effects of *R*_dark_ vertical gradients on understory LAI in FATES.
**Fig. S4** Effects of *R*_dark_ vertical gradients on canopy LAI in FATES.
**Fig. S5** Growth and maintenance respiration in FATES.
**Fig. S6** Effects of *R*_dark_ vertical gradients on global whole plant growth respiration in FATES.
**Fig. S7** Effects of *R*_dark_ vertical gradients on global whole plant maintenance respiration in FATES.
**Fig. S8** Canopy gradients of *R*_dark_ in the light demanding and shade‐tolerant PFTs for single site simulations.
**Table S1** Parameter ranges for two PFT single site perturbed parameter ensembles.
Please note: Wiley is not responsible for the content or functionality of any Supporting Information supplied by the authors. Any queries (other than missing material) should be directed to the *New Phytologist* Central Office.

## Data Availability

The data that support the findings of this study are openly available in the ESS‐DIVE archive at doi: 10.15486/ngt/2425967. This includes simulation outputs, parameter files and analysis scripts (Needham, 2025). Measurements of leaf traits and fluxes from vertical canopy profiles in Panama are available through the ESS‐DIVE archive (Lamour *et al*., 2021). See Lamour *et al*. (2022, 2023a) for details. All simulations were run using the ELM branch lnd/fates‐twostream with commit 5e483733fa on Ryan Knox's fork of E3SM and FATES branch jfn‐calibration‐rdark‐api33 with commit 77ac7623 on Jessica Needham's fork of FATES (https://github.com/rgknox/E3SM/tree/lnd/fates‐twostream; https://github.com/JessicaNeedham/fates/tree/jfn‐calibration‐rdark‐api33). These branches correspond to FATES API 33 (https://github.com/NGEET/fates/releases/tag/sci.1.71.0_api.33.0.0) with some additional changes described in the Supporting Information, which were merged into FATES main branch with later pull requests.
