# Supplementary material for: Vertical canopy gradients of respiration drive plant carbon budgets and leaf area index
Source: New Phytol. 2025 Feb 19;246(1):144–57. doi: 10.1111/nph.20423 (PMC11883058; doi:10.1111/nph.20423)
Supplement: Supplementary file 1 — Fig. S1 Effects of R dark vertical gradients on understory leaf allocation in FATES. Fig. S2 Effects of R dark vertical gradients on canopy leaf allocation in FATES. Fig. S3 Effects of R dark vertical gradients on understory LAI in FATES. Fig. S4 Effects of R dark vertical gradients on canopy LAI in FATES. Fig. S5 Growth and maintenance respiration in FATES. Fig. S6 Effects of R dark vertical gradients on global whole plant growth respiration in FATES. Fig. S7 Effects of R dark vertical gradients on global whole plant maintenance respiration in FATES. Fig. S8 Canopy gradients of R dark in the light demanding and shade‐tolerant PFTs for single site simulations. Table S1 Parameter ranges for two PFT single site perturbed parameter ensembles. Please note: Wiley is not responsible for the content or functionality of any Supporting Information supplied by the authors. Any queries (other than missing material) should be directed to the New Phytologist Central Office. [file NPH-246-144-s001.pdf]

New Phytologist Supporting Information

Article Title: Vertical Canopy Gradients of Respiration Drive Plant Carbon Budgets and Leaf Area Index

Authors: Jessica F. Needham, Sharmila Dey, Charles D. Koven, Rosie A. Fisher, Ryan G. Knox, Julien Lamour, Gregory Lemieux, Marcos Longo, Alistair Rogers, Jennifer Holm

Article acceptance date: 7 January 2025

FATES version

Changes to FATES that were added to API 33:

- Bug fix to fire intensity - https://github.com/NGEET/fates/pull/1180
- Canopy layers increased to 3 - https://github.com/NGEET/fates/pull/1198
- Daylength factor - https://github.com/NGEET/fates/pull/1161
- Alternative vertical scaling of Rdark - https://github.com/NGEET/fates/pull/1149
- Understory leaf lifespan - https://github.com/NGEET/fates/pull/1136

Table S1 Parameter ranges for two Plant Functional Type (PFT) single site perturbed parameter ensembles. PFTs; 0 - not a PFT specific parameter, 1 - Light demanding, 2 - Shade tolerant

parameter	min value	max value	PFT
fates_mort_disturb_frac	0.3	1	0
fates_mort_understorey_death	0	1	0
fates_leaf_slatop	0.009	0.015	2
fates_leaf_slatop	0.0096	0.018	1
fates_alloc_storage_cushion	2.1	3	2
fates_alloc_storage_cushion	1.8	2.8	1
fates_mort_bmort	0.0112	0.0168	2
fates_mort_bmort	0.0112	0.021	1
fates_wood_density	0.3	0.5	1

fates_mort_scalar_cstarvation	0.12	0.6	2
fates_mort_scalar_cstarvation	0.2	0.7	1
fates_turnover_branch	75	165	2
fates_turnover_branch	30	80	1
fates_recruit_seed_alloc	0.075	0.125	2
fates_recruit_seed_alloc	0.1	0.2	1
fates_recruit_seed_dbh_repro_threshold	60	100	2
fates_recruit_seed_dbh_repro_threshold	30	80	1
fates_maintresp_leaf_atkin2017_baserate	1.0536	2.1072	2
fates_maintresp_leaf_atkin2017_baserate	1.3170	2.1950	1
fates_leaf_vcmax25top	40	72	2

We used latin hypercube sampling to sample the parameters in table S1 and in addition resampled parameters for PFT 2 using the value of the parameter for PFT 1 as the upper or lower sampling bound in order to impose the following relationships:

- SLA is higher in the light demanding PFT.
- Background mortality is higher in the light demanding PFT.
- Carbon starvation mortality is higher in the light demanding PFT.
- Seed allocation is higher in the light demanding PFT.
- The base rate of Rdark is higher in the light demanding PFT.
- Allocation to storage is lower in the light demanding PFT.
- Allocation to reproduction starts at smaller sizes in the light demanding PFT.
- Branch turnover is faster in the light demanding PFT.

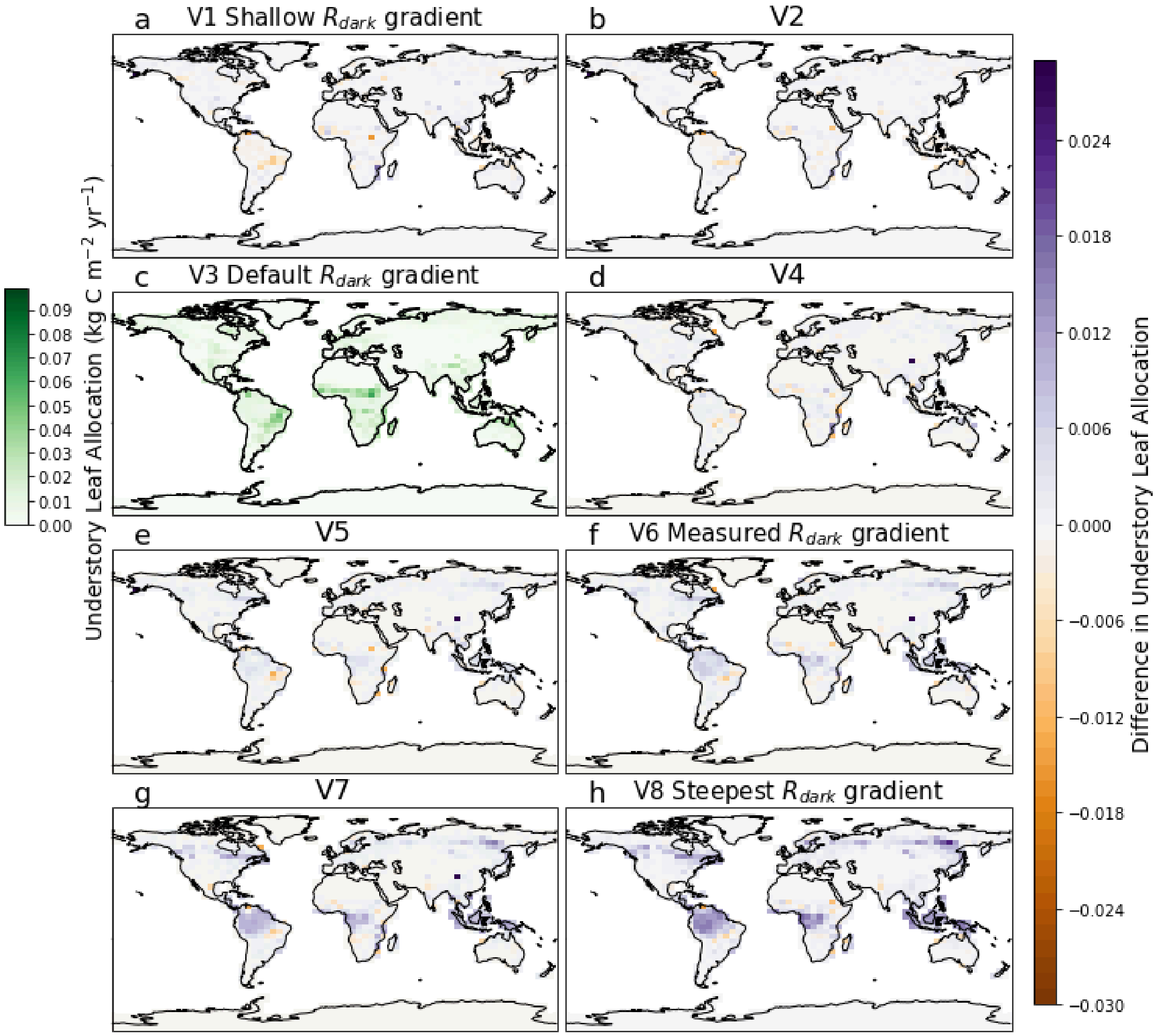

Figure S1. Effects of R_{dark} vertical gradients on understory leaf allocation in FATES. V3 is the default simulation in FATES in which R_{dark} and V_{cmax} are proportional through the canopy. The V3 panel shows the absolute values of allocation to leaves in the understory. Remaining panels show the difference between a given simulation and the default. Purple indicates that a given simulation had higher allocation of carbon to leaves in the understory than the default simulation (V3), whereas orange indicates lower allocation to leaves than the default simulation (V3).

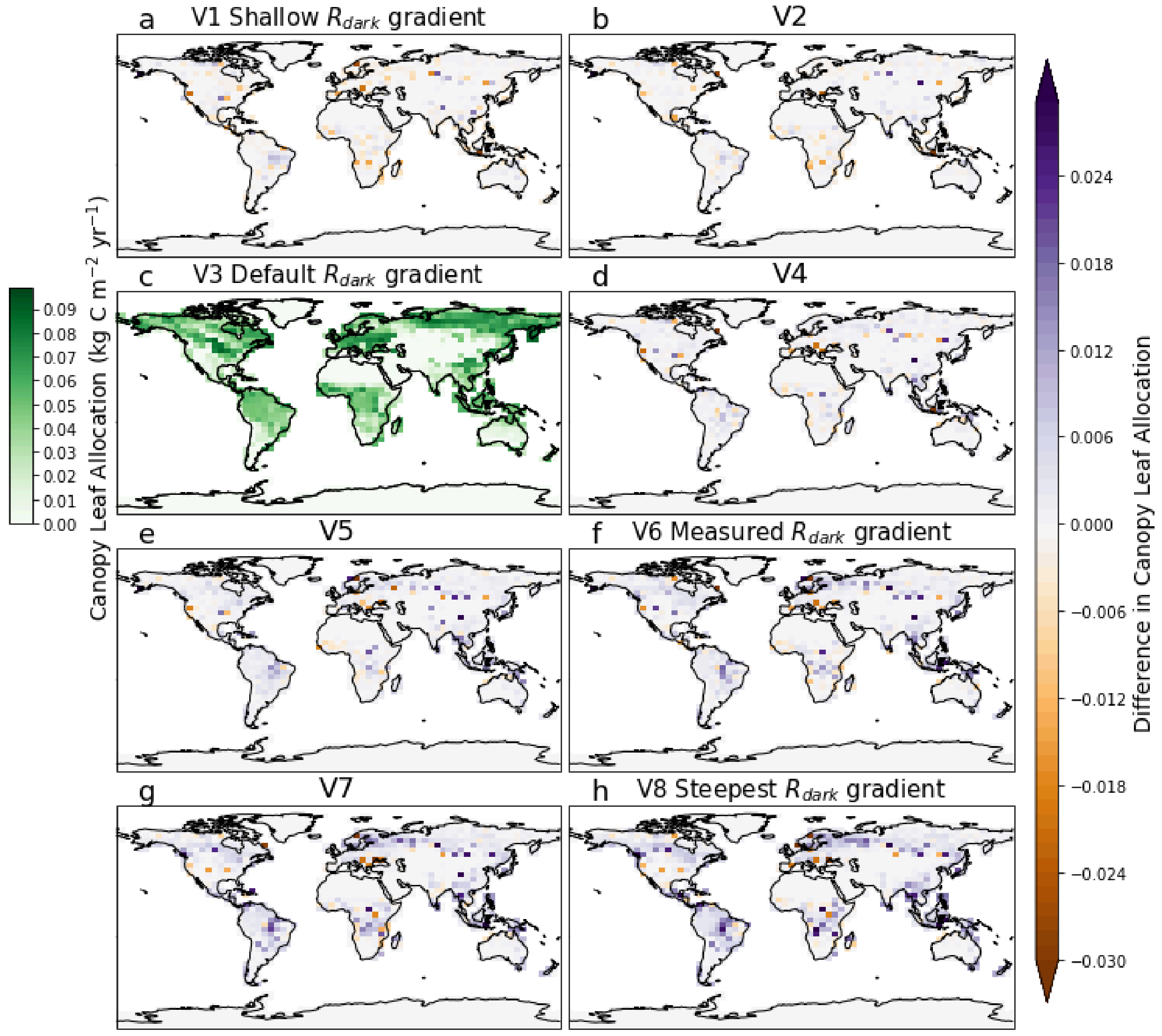

Figure S2. Effects of R_{dark} vertical gradients on canopy leaf allocation in FATES. V3 is the default simulation in FATES in which R_{dark} and V_{cmax} are proportional through the canopy. The V3 panel shows the absolute values of allocation to leaves in the canopy. Remaining panels show the difference between a given simulation and the default. Purple indicates that a given simulation had higher allocation of carbon to leaves in the canopy than the default simulation (V3), whereas orange indicates lower allocation to leaves than the default simulation (V3).

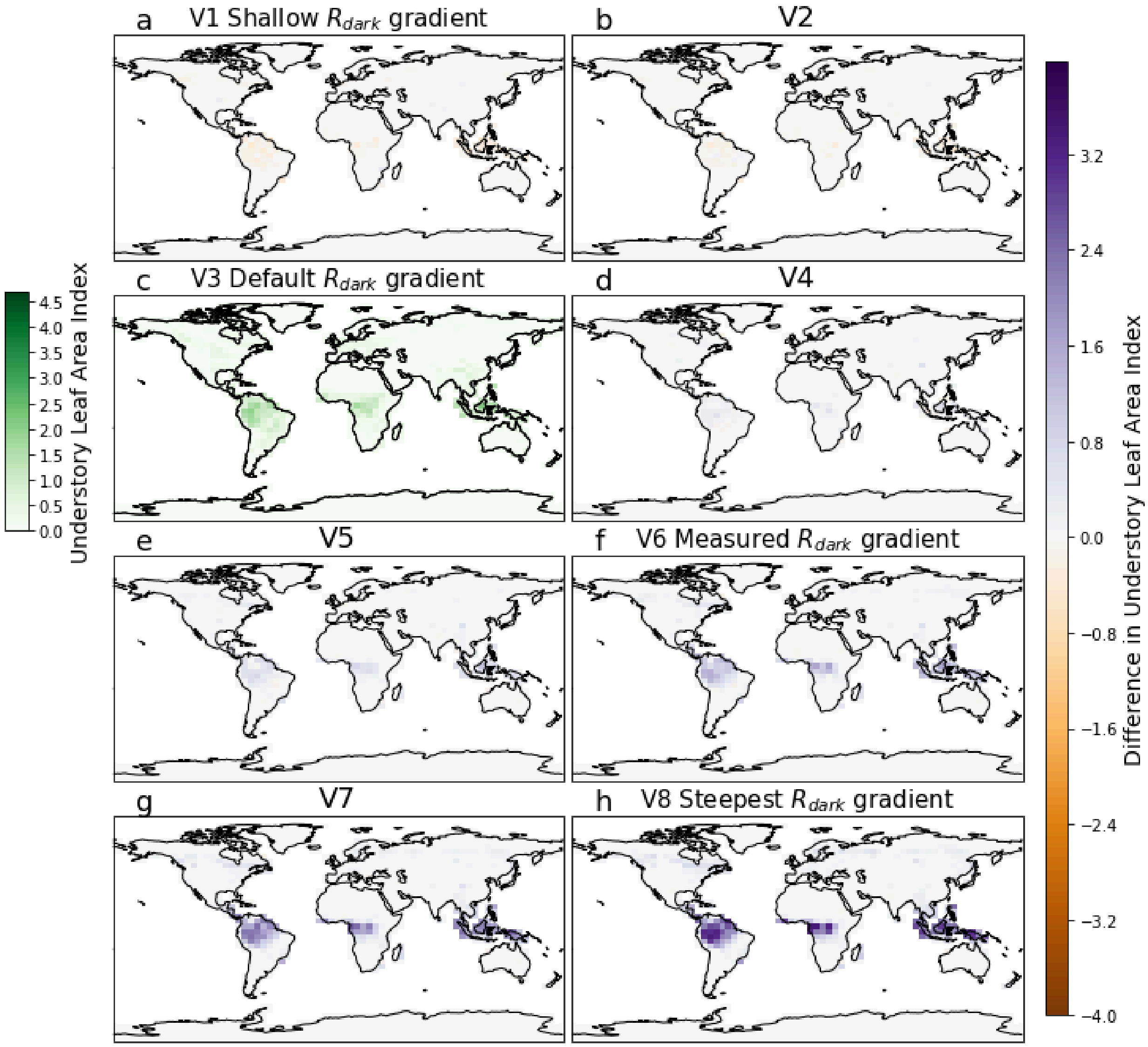

Figure S3. Effects of R_{dark} vertical gradients on understory LAI in FATES. V3 is the default simulation in FATES in which R_{dark} and V_{cmax} are proportional through the canopy. The V3 panel shows the absolute values of understory leaf area index (LAI). Remaining panels show the difference between a given simulation and the default. Purple indicates that a given simulation had higher understory LAI than the default simulation (V3), whereas orange indicates lower understory LAI than the default simulation (V3).

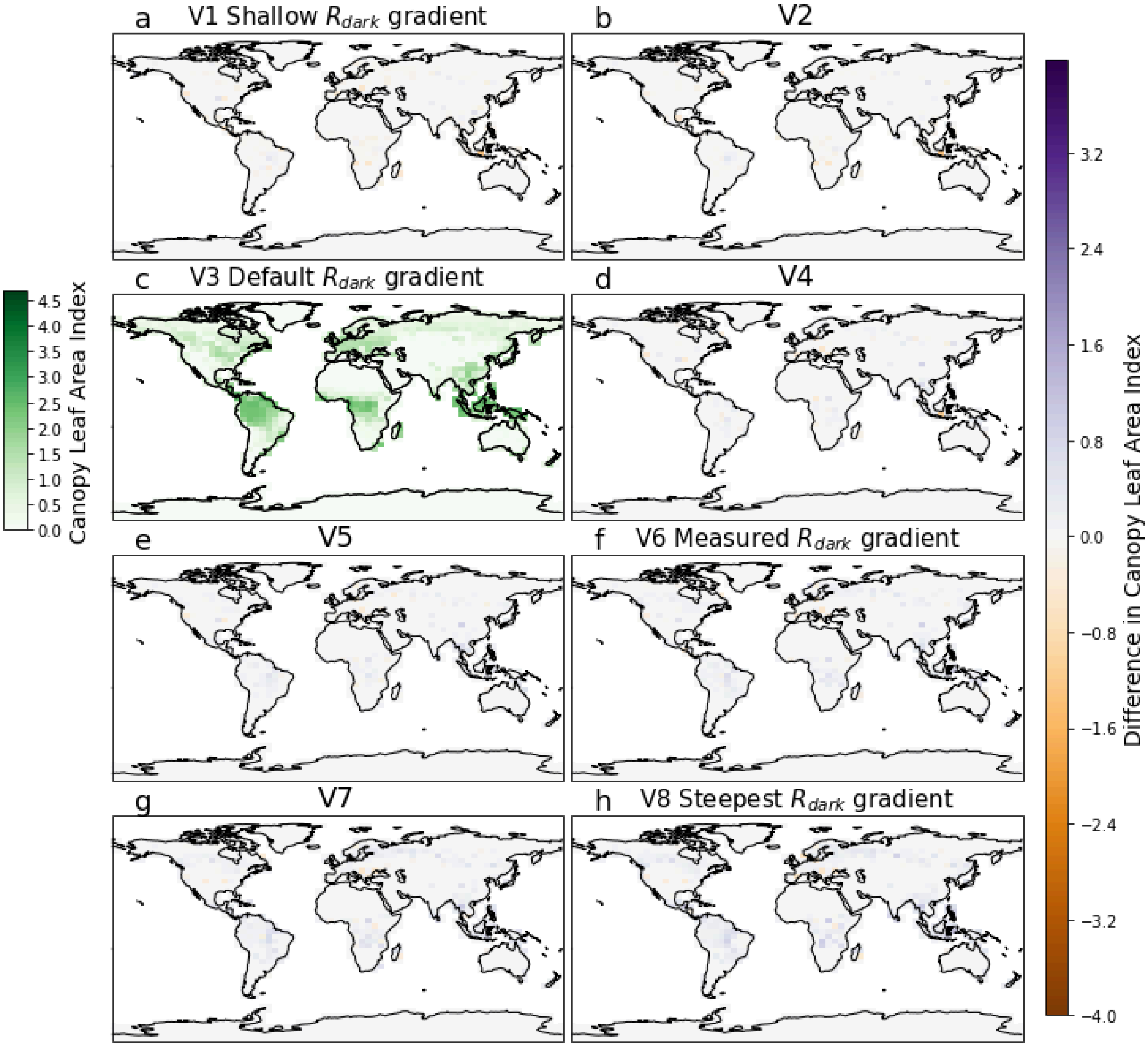

Figure S4. Effects of R_{dark} vertical gradients on canopy LAI in FATES. V3 is the default simulation in FATES in which R_{dark} and V_{cmax} are proportional through the canopy. The V3 panel shows the absolute values of canopy leaf area index (LAI). Remaining panels show the difference between a given simulation and the default. Purple indicates that a given simulation had higher canopy LAI than the default simulation (V3), whereas orange indicates lower canopy LAI than the default simulation (V3).

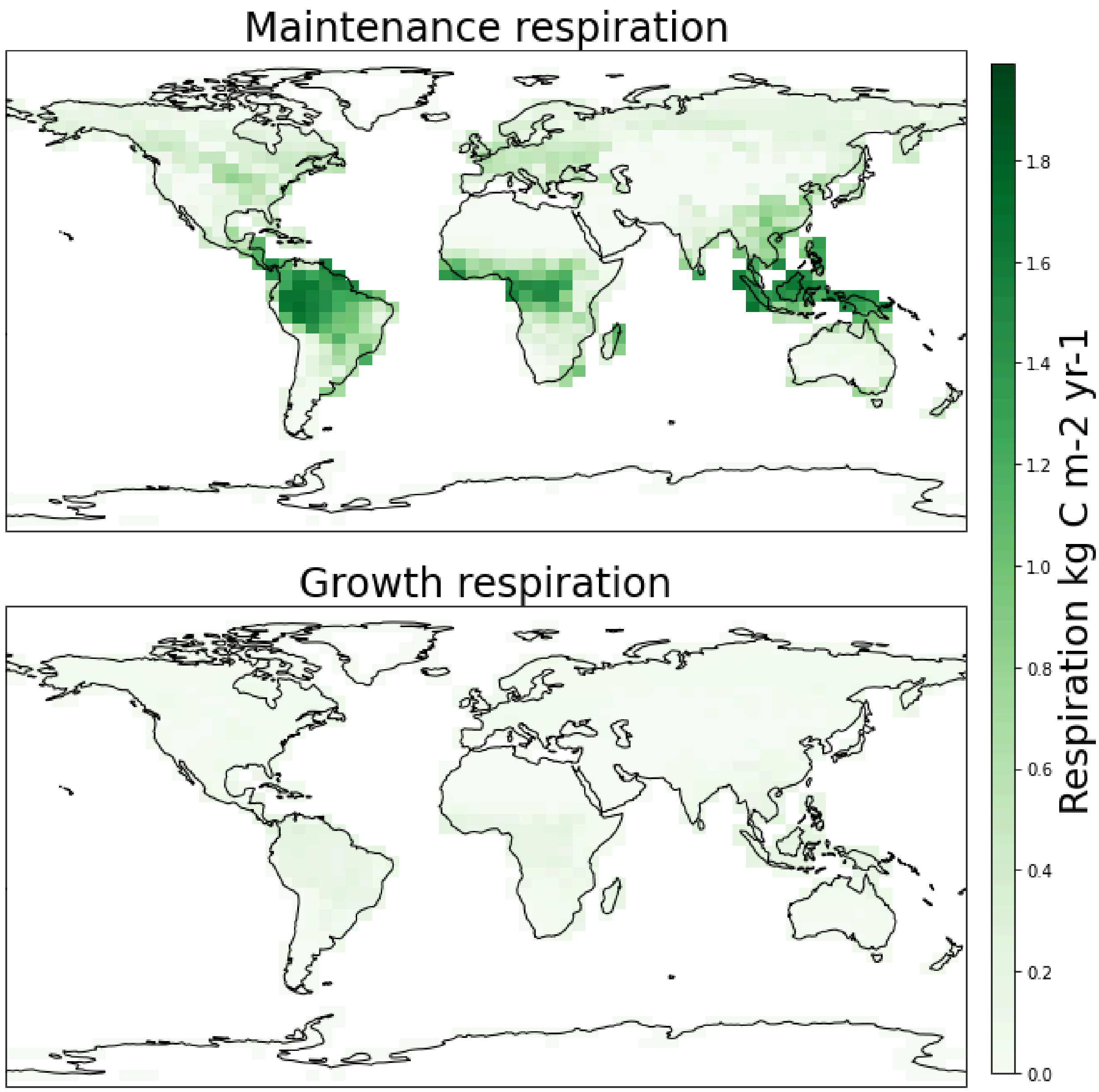

Figure S5. Growth and maintenance respiration in FATES. This figure shows the default parameterization in FATES, but regardless of the canopy gradient of Rdark, whole plant maintenance respiration is much higher than growth respiration.

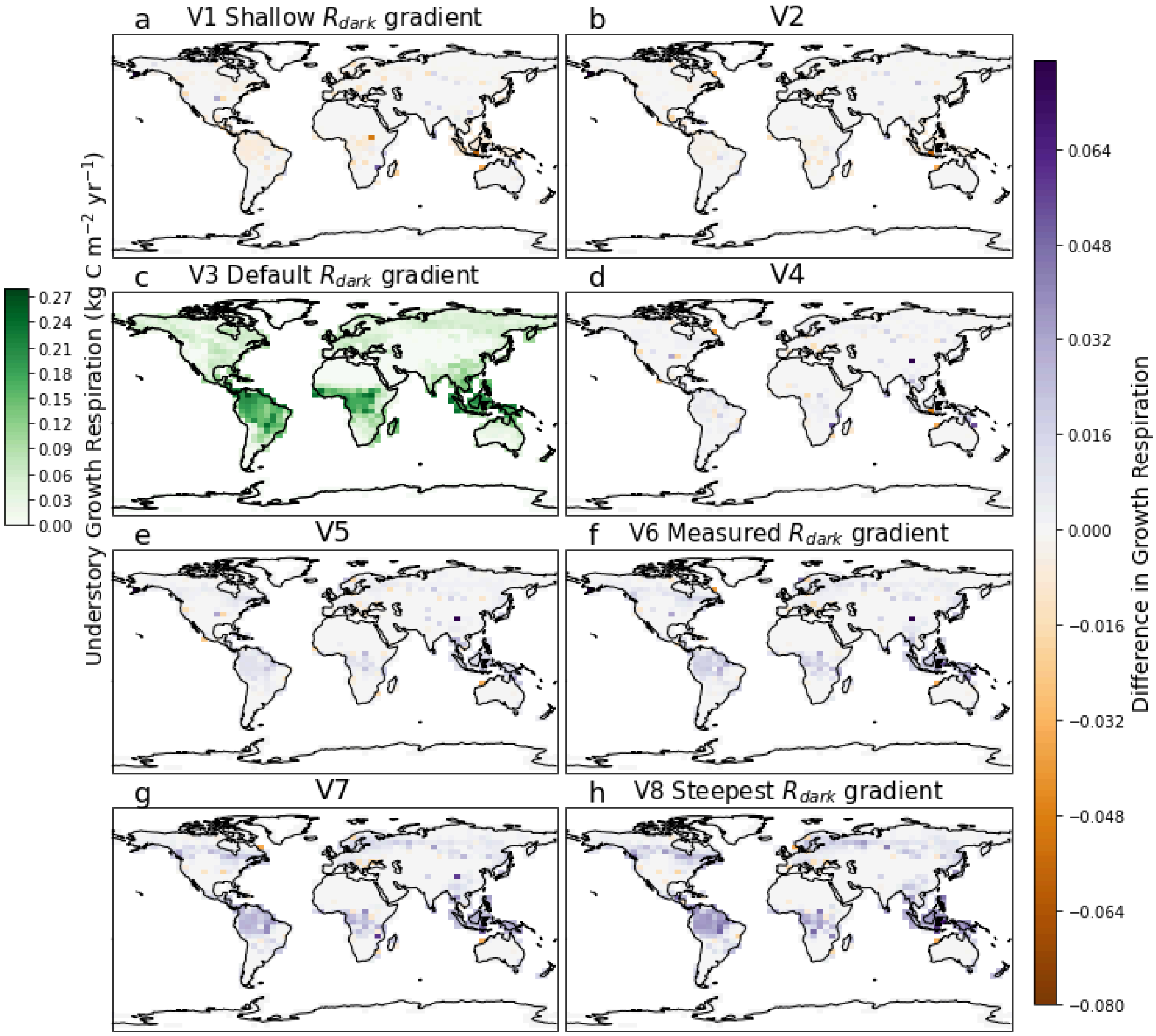

Figure S6. Effects of R_{dark} vertical gradients on global whole plant growth respiration in FATES. V3 is the default simulation in FATES in which R_{dark} and V_{cmax} are proportional through the canopy. The V3 panel shows the absolute values of growth respiration. Remaining panels show the difference between a given simulation and the default. Purple indicates that a given simulation had higher growth respiration than the default simulation (V3), whereas orange indicates lower growth respiration than the default simulation (V3).

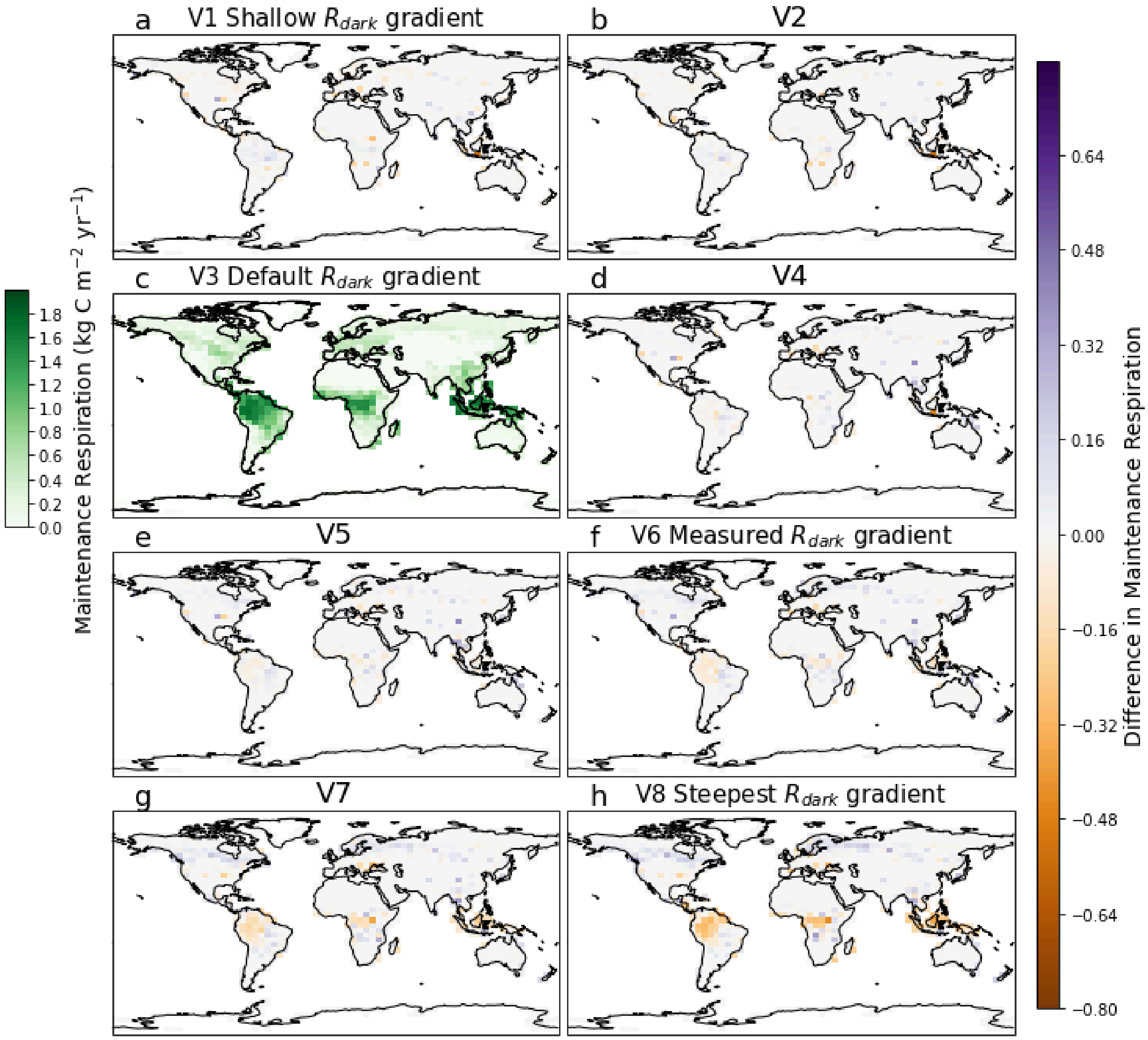

Figure S7. Effects of R_{dark} vertical gradients on global whole plant maintenance respiration in FATES. V3 is the default simulation in FATES in which R_{dark} and V_{cmax} are proportional through the canopy. The V3 panel shows the absolute values of maintenance respiration. Remaining panels show the difference between a given simulation and the default. Purple indicates that a given simulation had higher maintenance respiration than the default simulation (V3), whereas orange indicates lower maintenance respiration than the default simulation (V3).

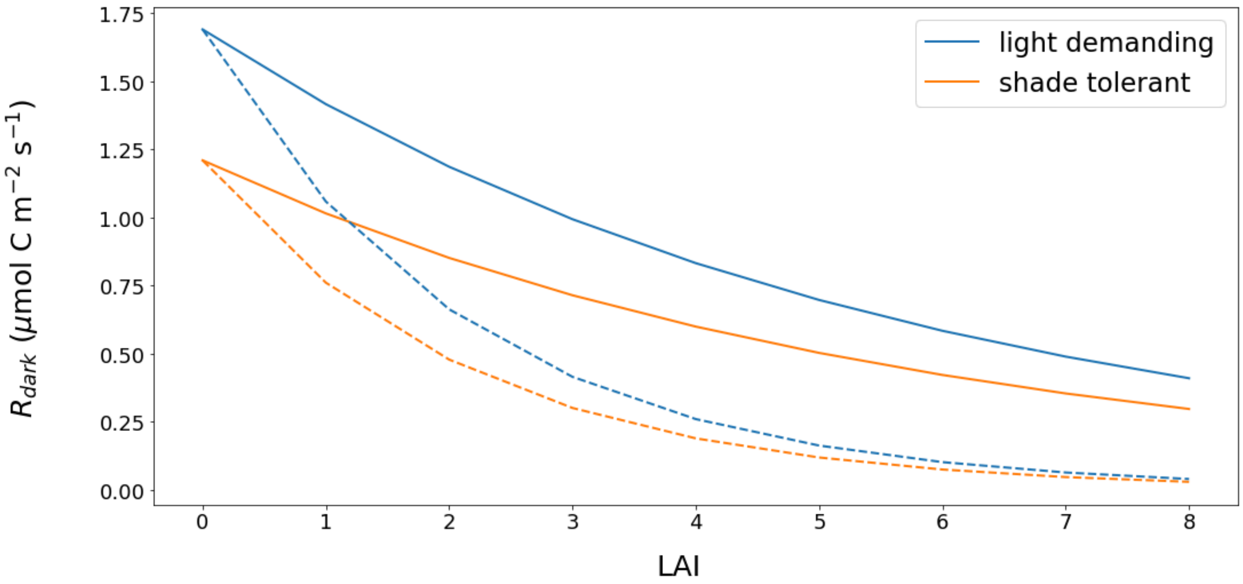

Figure S8. Canopy gradients of *R*$_{\text{dark}}$ in the light demanding and shade tolerant PFTs for single site simulations. Solid lines show gradients of *R*$_{\text{dark}}$ with the default parameterisation, dotted lines show gradients with the V7 simulation. Canopy gradients of *R*$_{\text{dark}}$ vary depending on Vcmax, SLA and the base *R*$_{\text{dark}}$ respiration rate. The figure here is for the simulation in which the shade tolerant PFT dominates with the default parameterisation.
